# Coconut oil/lauric acid-based nanodrug formulation: a multifunctional platform for enhancing cancer therapy and mitigating toxicity

**DOI:** 10.3389/fcell.2026.1800786

**Published:** 2026-05-11

**Authors:** Sorra Sandhya, Mohini Singh, Bani Kumar Jana, Joyeeta Talukdar, Debabrat Baishya, Gayatri Gogoi, Bikul Das, Bhaskar Mazumder

**Affiliations:** 1 Department of Pharmaceutical Sciences, Dibrugarh University (DU), Dibrugarh, Assam, India; 2 Department of Cancer Stem Cells and Infectious Diseases, Kavikrishna Laboratory, Indian Institute of Technology (IIT) Guwahati, Guwahati, Assam, India; 3 Department of Bioengineering and Technology, Gauhati University, Guwahati, Assam, India; 4 Department of Biochemistry, All India Institute of Medical Sciences (AIIMS), New Delhi, India; 5 Department of Pathology, Assam Medical College and Hospital, Dibrugarh, Assam, India; 6 Multidisciplinary Research Unit, Assam Medical College and Hospital, Dibrugarh, Assam, India; 7 Department of Experimental Therapeutics, Thoreau Laboratory for Global Health, M2D2, University of Massachusetts, Lowell, MA, United States

**Keywords:** anticancer drug, cancer, coconut kernel extract, coconut oil, lauric acid, nanodrug formulation, toxicity

## Abstract

The significant toxicity associated with anticancer drugs during traditional treatments, such as surgery, radiation, chemotherapy, and hormonal therapy, limits their therapeutic index by causing various adverse effects on healthy cells. Particularly, the clinical application of platinum (Pt)-based anticancer drugs (Pt-ADs), including cisplatin, carboplatin, and others, is challenging and severely hampered by intrinsic drug resistance, oxidative stress, and toxicity. Lipid-based nanodrug delivery systems have emerged as a promising solution to overcome these challenges, offering enhanced drug targeting, controlled release, and improved therapeutic indices. Among lipid-based carriers, coconut oil (CO) has attracted growing interest as a natural, biocompatible component for nanodrug formulations. Rich in medium-chain triglycerides (MCTs) and bioactive fatty acids such as lauric acid (LA), CO not only serves as an efficient solubilizing and stabilizing agent but also exhibits potential antioxidant, anticancer, antimicrobial, and immunomodulatory properties. Recently, we also found coconut kernel extract or a type of oil extracted from coconut kernel demonstrates considerable antioxidant and anticancer properties, as well as the capacity to diminish oxidative stress and the overexpression of the c-MYC proto-oncogene. Therefore, in this review, we explored the emerging role of CO/LA-based nanodrugs (CO/LA-NDs) in cancer therapy. We further focused on their anticancer effects with potential mechanistic insight followed by, a brief discussion of a few instances of CO/LA-based nanoformulation techniques’ capacity to mitigate systemic anticancer drug-mediated toxicity while enhancing anticancer efficacy, and the pharmacokinetic advantages of CO/LA-NDs in cancer research with an emphasis on pharmacological strategy to target cancer stem cells (CSCs). We also discussed how CO’s antioxidant properties might be used in the future to target CSCs and lessen Pt-AD-mediated toxicity. Therefore, by incorporating natural lipid systems into nanomedicine, CO/LA-based nanodrug (CO/LA-ND) formulations may offer a fresh, multifunctional platform for cancer treatment, enhancing efficacy and lowering side effects.

## 1 Introduction

Cancer remains the second leading cause of morbidity and mortality globally (Zafar et al., 2025), with 9.7 million deaths and 20 million new cases in 2022, despite significant progress in diagnosis and treatment (Bray et al., 2024). The American Cancer Society predicts that by 2050, there will likely be 35 million new cancer cases (Bray et al., 2024; Global Cancer Facts, 2024). Consequently, to improve cancer patients’ chances of survival, society needs to keep improving therapeutic strategies and broadening our understanding of tumor biology. Even with the advancements in hormonal, immuno, and targeted therapies, conventional cancer treatments like chemotherapy, surgery, and radiation remain the cornerstones of cancer treatment (Zafar et al., 2025; Yazbeck et al., 2022).

Chemotherapy uses powerful anticancer drugs like cisplatin, carboplatin, fluorouracil, *etc.*, to kill cancer cells, but their toxicity poses significant challenges in cancer management and treatment, including side effects like nausea, vomiting, cardiotoxicity, pulmonary toxicity, and renal toxicity (Yazbeck et al., 2022). Moreover, anticancer drugs often cause side effects like myelosuppression, oral mucositis, and alopecia due to harm to normal cells in the digestive system, hair follicles, and bone marrow (Pabla and Dong, 2012; Crawford et al., 2024; Lalla et al., 2008; Kaufman et al., 2025). Furthermore, despite being more selective, targeted therapies and immunotherapies can result in serious immune-related or off-target adverse reactions, including cytokine release syndrome, hepatotoxicity, and cardiotoxicity (Postow et al., 2018). Thus, anticancer drug-mediated toxicity remains a major challenge in successful cancer therapy, often limiting the effectiveness of treatment and significantly impacting patients’ quality of life (Yazbeck et al., 2022; Lee et al., 2023). Particularly, intrinsic drug resistance, oxidative stress, and toxicity significantly restrict the clinical use of platinum (Pt)-based anticancer drugs (Pt-ADs), such as cisplatin, carboplatin, oxaliplatin, *etc.*, (Zhou et al., 2020; Zhang et al., 2022; Cauli, 2021; Tolan et al., 2016; Go and Adjei, 1999; Woynarowski et al., 2000). Several studies have been carried out to minimize the toxicity and oxidative stress brought on by Pt-ADs, which are associated with severe adverse effects, particularly neurotoxicity (Staff et al., 2019), ototoxicity (Babolmorad et al., 2021), myelosuppression (Zhang et al., 2022; Das et al., 2008), and dose-limiting nephrotoxicity (Oun et al., 2018; Schoch et al., 2021). Thus, anticancer drug-mediated toxicities not only pose risks to patient safety but can also necessitate dose reductions or treatment discontinuation, compromising therapeutic outcomes.

Additionally, cancer stem cells (CSCs), a tiny subgroup of cancer cells marked by stemness phenotype, i.e., self-renewal and differentiation potential (Baishya et al., 2025; Das et al., 2019), also play critical role in anticancer drug-mediated toxicity. These CSCs exhibit inherent drug resistance to conventional therapies, driven by mechanisms such as overexpression of drug efflux pumps (e.g., ATP-binding cassette (ABC) transporter), enhanced DNA repair capabilities, evasion of apoptosis, and maintenance of a quiescent/dormant state (Dean et al., 2005), contributing to cancer relapse and metastasis. Table 1 illustrated the possible link between CSCs and anticancer drug-mediated toxicity. Therefore, in an effort to eliminate CSCs, higher doses or more aggressive combinations of anticancer drugs are often employed, which can cause significant toxicity to normal tissues, particularly those containing normal stem cells that share similar signalling pathways (such as WNT, NOTCH, and HEDGEHOG) with CSCs (Zhou et al., 2009). To address these issues, the modification of anticancer drugs into nanotechnology-based delivery systems has come out as a favorable strategy in cancer research. This strategy enables targeted drug delivery that can strengthen therapeutic effectiveness while while reducing unintentional harm to healthy tissues (Ammar et al., 2025; Chehelgerdi et al., 2023). Nanoparticles can be formulated to categorically target CSCs by conjugating them with ligands or antibodies against CSC surface markers (e.g., CD44, CD133, or EpCAM), thereby enhancing drug accumulation in CSC-rich regions while sparing normal tissues (Roszkowski et al., 2024; Liu et al., 2023; Ertas et al., 2021). Additionally, nano-formulations can upgrade the solubility, stability, and bioavailability of chemotherapeutic agents, allow for controlled drug release, and reduce systemic exposure, which collectively contributes to lower toxicity profiles (Danhier et al., 2010). Thus, nanotechnology offers a dual advantage: upgrading the therapeutic index of anticancer drugs and overcoming the limitations posed by CSC-associated drug resistance and toxicity.

**TABLE 1:** The potential association between cancer stem cells (CSCs) and anticancer drug-mediated toxicity.

Aspect	Explanation based on evidence
Drug resistance	CSCs can resist conventional chemotherapy due to their ability to activate robust DNA repair, exhibit elevated drug efflux pump levels, and enter a state of quiescence. This resistance allows CSCs to survive therapy that kills the majority of tumor cells, leading to tumor relapse and metastasis (Dean et al., 2005; Phi et al., 2018; Li et al., 2020a; Begicevic and Falasca, 2017; Li and Bhatia, 2011).
Toxicity implications	To eradicate resistant CSCs, higher dosages of aggressive anticancer drugs are needed, which raises the systemic toxicity to healthy tissues. This occurs because CSCs are frequently unable to be eliminated by conventional therapy like chemotherapy and radiation, which allows them to spread and cause tumor recurrence (Phi et al., 2018; Rezayatmand et al., 2022).
Shared markers with normal stem cells	CSCs as well as normal stem cells share common markers like CD44, aldehyde dehydrogenase (ALDH), and the expression of genes implicated in the WNT, JAK-STAT and NOTCH, and HEDGEHOG signalling pathways (Zhou et al., 2009; Bourguignon et al., 2008; Verstappe and Berx, 2023; Januchowski et al., 2013). Drugs that target CSCs may unintentionally damage healthy stem cells, resulting in toxic side effects like mucositis, neurotoxicity, and bone marrow suppression (Zeien et al., 2022; Ramos et al., 2017).
Microenvironment protection	CSCs reside in hypoxic niche (with low oxygen and low pH) within tumor microenvironment (TME), which is primarily consist of fibroblasts and endothelial, mesenchymal, and immune cells, has a major impact on drug resistance (Bhuyan et al., 2022; Prieto-Vila et al., 2017). This niche helps protect CSCs from many conventional cancer treatments, making them a significant challenge for achieving a complete and lasting cure. Strategies to overcome this (e.g., modifying TME) can increase off-target toxicity (Ramos et al., 2017).
CSC-driven Heterogeneity	Tumors with a high content of CSCs exhibit greater genetic and epigenetic diversity, which is a major reason for variable drug responses and unpredictable toxicity profiles in patients. This high level of variability is driven by the dynamic and adaptable nature of CSCs, allowing them to evade therapy and drive tumor recurrence and metastasis (Phi et al., 2018; Rezayatmand et al., 2022; Thankamony et al., 2020).

Among the various nanocarrier platforms, lipid-based nanodrug delivery systems have gained considerable attention due to their biocompatibility, capacity to encapsulate both hydrophilic and hydrophobic drugs, capability to release drugs s in a regulated way, and potential to target CSCs in the tumor microenvironment (TME), indicating improved therapeutic indices (Yao et al., 2020; Giordano et al., 2024; Samad et al., 2025). Lipid-based carriers, including liposomes and solid lipid nanoparticles, are well established delivery methods; however, those employing coconut oil (CO) and its major derivatives lauric acid (LA) for drug encapsulation offer distinct biological advantages (Dhanya et al., 2023; Pornpattananangkul, 2016; Ali Alghamdi et al., 2024). CO serves as a promising lipid matrix for nanodrug formulation due to its high medium-chain triglyceride (MCT) content, particularly LA, which demonstrates rapid enzymatic hydrolysis, improved intestinal permeability, and preferential transport *via* the portal vein compared to long-chain lipids, thereby enhancing drug bioavailability and facilitating lymphatic absorption (Dhanya et al., 2023; Jadhav and Annapure, 2023). This natural lipid source can be processed into solid lipid nanoparticles (SLNs) and nanostructured lipid carriers (NLCs) to improve drug delivery, protect encapsulated drugs from enzymatic degradation and other environmental factors, and achieve sustained release, though challenges with stability and particle size control need to be managed. Furthermore, CO possesses Generally Recognized as Safe (GRAS) status (Burnett et al., 2011), excellent biocompatibility, low toxicity, and cost-effective scalability. These intrinsic metabolic and physicochemical characteristics suggest that CO/LA-based systems may provide a simplified yet functionally advantageous alternative lipid matrix for enhancing oral bioavailability and membrane interaction compared with conventional structured lipid nanocarriers.

Moreover, CO may possess inherent anticancer and immunomodulatory properties (Verma et al., 2019; Bose et al., 2025). In line with these finding we also observed that coconut kernel extract (CKE) or a type of oil obtained from coconut kernel possesses significant antioxidant capacity and prevented tumor formation in carcinogen-induced skin cancer models by mitigate oxidative stress and c-MYC proto-oncogene overexpression (Sandhya Sorra et al., 2019; Sandhya, 2017; Sandhya et al., 2016; Sandhya et al., 2024). Additionally, we found that LA contributes significantly to the anticancer potential of CKE. (Sandhya et al., 2024). Furthermore, overexpression of the c-MYC proto-oncogene is a central cornerstone in cancer progression, increases pluripotency and tumorigenicity (Sandhya et al., 2024; Knoepfler, 2009; Greulich et al., 2000). This gene causes cells to grow aggressively by interfering with cell division, proliferation, and apoptosis (Sandhya et al., 2024; Dhanasekaran et al., 2022; Kaczmarek et al., 1985; Zindy et al., 1998; Amati and Land, 1994; Schlagbauer-Wadl et al., 1999; Marconi et al., 2022; Kreuzaler et al., 2019; Zimmerli et al., 2022). Researchers are therefore looking into this gene as a potential target for cancer treatment (Duffy et al., 2021; Wang et al., 2021; Llombart and Mansour, 2022; Chen et al., 2018). Our previous findings suggested that CSCs maintain self-renewal capacity by c-MYC-mediated HIF-2α stemness pathway *via* NANOG and SOX-2 (Das et al., 2019). Therefore, utilization of CO as lipid matrix for encapsulating anticancer drug may enhance it therapeutic efficacy by showing dual effect in the treatment of cancer: the anticancer drug targets cancer cells, while antioxidant effect of CO shields normal cells from the drug toxicity without protecting cancer cells. This strategy greatly lowers toxicity while simultaneously increasing therapeutic efficacy, making nanomedicine an essential tool for improving cancer Therapy.

Therefore, in this review we explored the emerging role of CO/LA-based nanodrugs (CO/LA-NDs) in cancer therapy, focusing on their anticancer effects with potential mechanistic insight, a brief discussion of a few instances of CO/LA-based nanoformulation techniques’ capacity to mitigate systemic anticancer drug-mediated toxicity while enhancing anticancer efficacy, and the pharmacokinetic advantages of CO/LA-NDs in cancer research with an emphasis on pharmacological strategy to target CSCs. We also discussed how CO’s antioxidant properties might be used in the future to target CSCs and lessen Pt-AD-mediated toxicity. Incorporating natural lipid systems into nanomedicine allows for CO/LA-based nanodrug (CO/LA-ND) formulations to become a multifunctional platform for cancer treatment, improving efficacy and reducing side effects. Thus, this review integrates insights from nanotechnology, oncology, and natural product chemistry, presenting CO/LA-ND delivery systems as a synergistic approach to enhance cancer treatment outcomes.

## 2 Anticancer effects of coconut oil (CO) and its derivatives

**FIGURE 1:**
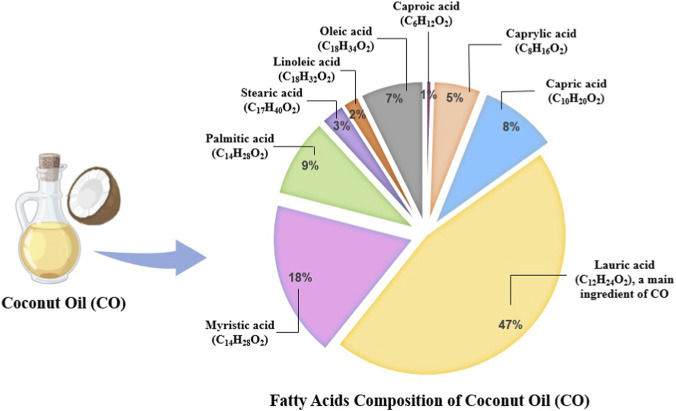
A pie diagram depicts the fatty acid content of coconut oil (CO), emphasizing the approximate percentages of Caproic, Caprylic, Capric, Lauric, Myristic, Palmitic, Stearic, Linoleic, and Oleic acids.

CO, a natural lipid extract derived primarily from the kernel of *Cocos nucifera* L., exhibits notable anticancer properties attributed to its unique fatty acid composition (Figure 1), particularly MCTs such as LA, capric acid, and caprylic acid. It comprises over 85%–90% saturated fatty acids, including MUFA and polyunsaturated (PUFA) fatty acids, along with 9% unsaturated fatty acids (Begicevic and Falasca, 2017). Among the saturated fraction, LA (*C*_12_) is usually the most abundant (often 40%–50%), followed by myristic (*C*_14_), palmitic (*C*_16_), caprylic (*C*_8_) and capric (*C*_10_) in smaller amounts (Barlina et al., 2022; Ghosh et al., 2014). Recent years have seen an increase in interest in the potential anticancer or antiproliferative properties of CO and its constituent fatty acids. Each fatty acids found in CO have individual anticancer effects on various cancer types, including oral, lung, neuroblastoma, hepatocellular, Ishikawa endometrial cancer, colon, breast, and pancreatic ductal adenocarcinoma illustrated in Table 2. But because of their strong antioxidant and anticancer capacities, coconut-based dietary product, particularly virgin CO (VCO) and its primary ingredient LA, has been extensively studied for its cytotoxic potential against various cancer cell lines.

**TABLE 2:** List of fatty acids found in coconut oil (CO) and their anticancer effects against different types of cancer.

Sl No.	Fatty Acids and its IUPAC name	Approximate % in coconut oil* (Bose et al., 2025; Firestone, 2006; MacDonald et al., 2018; Sabahannur and Alimuddin, 2022)	Anticancer effects/Mechanisms of action	Cancer types studied
1	Caproic acid (Hexanoic acid- C_6_H_12_O_2_)	∼0%–0.6%	- All three fatty acids, Capric, caprylic, and caproic acids, inhibits cancer cell proliferation by down regulating cell cycle control genes such as CDK2, CDK4, CKSIb, CCNA2, CCND1 expression and up regulating apoptosis-related genes like Gadd45α, NR4A1, P21 (Narayanan et al., 2015)]. **-** Capric acid reduces tumor growth, induces apoptosis and suppresses the c-Met phosphorylation (Yang et al., 2023).	Human colorectal carcinoma [HCT-116 (Narayanan et al., 2015), and HCT-15 (Sheela et al., 2019)], human skin epidermoid carcinoma [A-431 (Narayanan et al., 2015)], breast cancer [MDA-MB-231 (Narayanan et al., 2015)], hepatocellular carcinoma (HCCLM3 and HepG2 (Sheela et al., 2019; Yang et al., 2023])
2	Caprylic acid (Octanoic acid- C_8_H_16_O_2_)	∼4%–6%		
3	Capric acid (Decanoic acid- C_10_H_20_O_2_)	∼5%–10%		
4	Lauric Acid (Dodecanoic acid- C_12_H_24_O_2_)	∼45%–50%	-Induces apoptosis *via* oxidative stress (↑ ROS), mitochondrial damage (Bose et al., 2025; Sheela et al., 2019; Mori et al., 2025; Fauser et al., 2013; Lappano et al., 2017) -Decreases cell viability vis downregulating of EGFR signaling pathways (Bose et al., 2025; Sheela et al., 2019). -Promotes the phosphorylation of EGFR, ERK, and c-JUN, and raises the expression of c-FOS (Lappano et al., 2017). -Arrests cell cycle (S and G2/M), reduces glutathione, increases ROS (Bose et al., 2025; Fauser et al., 2013) -Alters cancer cell metabolism (inhibits oxidative phosphorylation), modulates immune responses (Bose et al., 2025; Mori et al., 2025). -Overcomes gemcitabine (GEM) resistance *via* decreasing mitochondrial ROS and increasing stemness associated to mitochondrial damage (Takagi et al., 2023).	Human colon cancer (Caco-2 [Fauser et al., 2013), IEC-6 (Fauser et al., 2013), CT26 (Mori et al., 2025), HT29 (Mori et al., 2025) and HCT-15 (Sheela et al., 2019)], human hepatocellular carcinoma [HepG2 (Sheela et al., 2019)], Breast cancer [SkBr3 (Lappano et al., 2017)], Ishikawa endometrial cancer cells (Lappano et al., 2017) and pancreatic ductal adenocarcinoma (MIA-PaCa-2, PANC-1 and Capan-2 (Takagi et al., 2023)
5	Myristic acid (Tetadecanoic acid- C_14_H_28_O_2_)	∼16%–20%	- Much less well studied in terms of direct anticancer action, however higher circulating levels of myristic acid have been reported to be positively linked with risk of both estrogen receptor positive (ER+) and estrogen receptor negative (ER-) breast cancer (Matta et al., 2022)	Breast cancer patients (Matta et al., 2022)
6	Palmitic acid (Hexdecanoic Acid- C_14_H_28_O_2_)	∼8%–10%	-Inhibits metastasis in prostate cancer cells by inhibiting the PI3K/AKT pathway, inducing G1 phase arrest, and upregulating p27 [(Zhu et al., 2021)]. -By increasing CD36 expression in colon cancer cells, palmitic acid triggers ER stress, ER calcium release, and transferrin-dependent ferroptosis (Kuang et al., 2023). -By suppressing the expression of p-STAT3, p-JAK2, N-cadherin, and vimentin, palmitic acid reduces cell growth, metastasis, and promotes apoptosis (Yu et al., 2023).	Prostate cancer [PC3 and DU145 (Zhu et al., 2021)], colon cancer [HT29, HCT116, SW480, SW620 (Kuang et al., 2023)], and gastric cancer [MGC-803, BGC-823, and SGC-7901 (Yu et al., 2023)]
7	Stearic acid (Octadecanoic acid- C_17_H_40_O_2_)	∼2%–3%	-Induces apoptosis *via* decreasing protein kinase C, which stops caspase-3 activation (Evan et al., 2009). -Lowers the incidence of carcinogen-induced breast tumor burden by decreasing cell cycle progression and suppressing Rho activation and expression (Li et al., 2011).	Human breast cancer (Hs578t and MDA-MB-231 (Evan et al., 2009; Li et al., 2011])
8	Linoleic acid (cis, cis-9,12-Octadecadienoic acid- C_18_H_32_O_2_)	∼1%–2%	- causes cell death through the mitochondrial apoptotic pathway (Lu et al., 2010).	Colorectal cancer [LOVO and RKO (Lu et al., 2010)]
9	Oleic acid (Cis-9-Octadecenoic acid- C_18_H_34_O_2_)	∼5%–8%	- induces autophagy by inducing senescence, lowering P-ERK, and reducing the anti-apoptotic proteins c-FLIP and BCL-2 (Giulitti et al., 2021)]. -Oleic acid induces apoptosis and autophagy in oral squamous cell carcinoma cells by causing cell cycle G0/G1 arrest and apoptosis, decreasing Cyclin D1 and BCL-2 expression, increasing p53 and cleaved caspase-3 expression, and blocking the AKT/mTOR pathway (Jiang et al., 2017).	Hepatocellular carcinoma [HCC (Giulitti et al., 2021)] and oral cancer [UM1 and CAL27 (Jiang et al., 2017)]

Abbreviations: AKT, A serine/threonine protein kinase also known as protein kinase B (PKB); BCL-2, B-cell leukemia/lymphoma 2 protein; CCNA2, Cyclin A2; CCND, Cyclin D; CDK2, Cyclin-dependent kinase 2; CDK4, Cyclin-dependent kinase 4; c-FLIP, cellular FLICE-inhibitory protein; c-JUN and c-FOS, Proto-oncogenes; CKSIb, CDC 28 protein kinase 1B; EGFR, Epidermal growth factor receptor; ER, Estrogen receptor; ERK, Extracellular signal-related kinases; Gadd45α, Growth Arrest and DNA Damage-inducible 45-alpha; GCLC, Glutamate-cysteine ligase catalytic subunit; HCC, hepatocellular carcinoma; HO-1, Heme oxygenase-1; IL6, Interleukin-6; JAK, Janus kinase 2; NR4A1, Nuclear receptor subfamily 4 group A member 1; iNOS, Inducible nitric oxide synthase; mTOR, mammalian target of rapamycin; NQO1, NAD(P)H Quinone Dehydrogenase 1; P21, Cyclin-dependent kinase inhibitor 1; p27 and p53, Tumor suppressor gene; PI3K, Phosphatidylinositol 3-kinase; ROS, Reactive Oxygen Species; STAT3, signal transducer and activator of transcription 3; TNFα, Tumor Necrosis Factor alpha

VCO and LA demonstrate potential in cancer therapy by directly targeting cancer cells and alleviating the toxic side effects of conventional chemotherapeutics, suggesting a dual role in reducing toxicity (Bose et al., 2025; Famurewa et al., 2017; Anthonia Chiamaka et al., 2023; Ramya et al., 2022). According to studies, VCO and LA have demonstrated strong anti-proliferative effects against a variety of cancer cell lines, including those from breast, lung, colorectal, colon, liver, oral, and neuroblastoma cells. They also suggested that VCO and LA can trigger apoptosis and improve the effects of chemotherapy (Verma et al., 2019; Bose et al., 2025; Ramya et al., 2022; Kamalaldin et al., 2013; Law et al., 2014; Sheela et al., 2019; Mori et al., 2025). By inducing oxidative stress and cell cycle arrest, the LA is believed to be a major active ingredient in VCO that gives these anti-cancer effects (Bose et al., 2025; Sandhya et al., 2024; Sheela et al., 2019). A study based on comparison between VCO, fractionated CO (FCO), and processed CO (PCO) confirms that differences in fatty acid composition are associated with differences in anticancer efficacy. For instance, 20% VCO significantly cytotoxically affected HepG2 (liver cancer) cells due to elevated levels of medium-chain fatty acids and LA, while high concentrations of PCO (approximately 80%) were more effective against oral cancer cells (Verma et al., 2019). Another study, demonstrated that compared to refined CO, VCO and crude CO caused more growth inhibition, nuclear fragmentation, and disruption of the mitochondrial membrane potential in the neuroblastoma cell line SH SY5Y, suggesting that oil purity or processing is crucial for anticancer effectiveness (Ramya et al., 2022). Crucially, we also found that in carcinogen induced skin cancer model, crude extract or CKE showed that administration reduces oxidative stress, lowers lipid peroxidation, enhances endogenous antioxidant enzyme activities (glutathione, catalase, superoxide dismutase), and downregulates c-MYC proto-oncogene expression—leading to reduced tumor incidence (Sandhya et al., 2024). Our findings also demonstrated that LA is a key component of CKE as per High performance liquid chromatography (HPLC) analysis and LA treatment also demonstrated anticancer properties against skin cancer caused by DMBA/TPA (Sandhya et al., 2024). In line with our findings other studies also showed that LA induces dose-dependent cytotoxicity in colon cancer (HCT-15), hepatocellular carcinoma (HepG2), and other cancer lines, often *via* apoptotic morphologic changes (Sheela et al., 2019). In HCT-15 colon cancer cells, LA treatment (30–50 μg/mL) downregulated epidermal growth factor receptor (EGFR) expression, which is a known driver of proliferation in many cancers (Sheela et al., 2019).

Moreover, it has been demonstrated that mannooligosaccharides derived from coconut meal (CMOSs) inhibit the growth of colorectal cancer (HCT116) cells, enhance caspase activation (8, 9, 3/7), produce reactive oxygen species (ROS), and inhibit migration and angiogenesis *in vitro* with less harm to healthy cells (Pason et al., 2024). Even in several hepatic, breast, and colon cancer cell lines, Tender Coconut Water (TCW) exhibits anticancer effects by decreasing cell viability, causing cell cycle arrest in the S phase, suppressing EMT (epithelial to mesenchymal transition) markers (like increased E cadherin and decreased N cadherin), and blocking AKT and ERK signalling without influencing healthy epithelial cells (Lakshmanan et al., 2025). These studies therefore suggested that CO might have two functions in lowering toxicity during cancer treatment: it may target cancer cells by inducing apoptosis, and it may also shield healthy cells from anticancer medications by cytoprotecting them (Bose et al., 2025; Ramya et al., 2022). Advanced formulations like nanoemulsion can further enhance these effects.

### 2.1 Mechanism of action of coconut oil (CO) and lauric acid (LA) in cancer therapy

The major mechanistic pathways by which CO and LA exert anticancer effects are multifactorial, involving generation of ROS and induction of oxidative stress, apoptosis induction *via* multiple signaling cascades, cell cycle arrest and inhibition of proliferation, modulation of cancer-related signalling pathways, metabolic reprogramming and lipid metabolism impacts, and selectivity toward cancer vs. normal cells and potential for synergism.

#### 2.1.1 ROS production and oxidative stress induction

**FIGURE 2:**
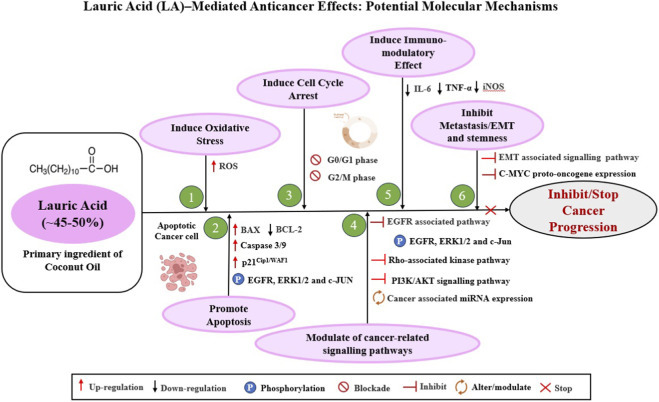
Schematic overview of the molecular mechanisms underlying the anticancer effects of lauric acid (LA), a major component of coconut oil (CO). Based on evidence, a representative image illustrates the potential mechanism of action of LA that enhances inhibition of cancer progression. **(1)** Induce oxidative stress by generating ROS levels in cancer cells (Bose et al., 2025; Sheela et al., 2019; Mori et al., 2025; Fauser et al., 2013; Lappano et al., 2017). **(2)** Promote apoptosis by up-regulating the cyclin-dependent kinase inhibitor p21^Cip1/WAF1^ in a p53-independent manner (Lappano et al., 2017), phosphorylating EGFR, ERK, and c-JUN signalling pathways (Bose et al., 2025; Sheela et al., 2019; Lappano et al., 2017), and causing mitochondrial damage by changing the activities of BAX, BCL-2, and Caspase 3/9 (Bose et al., 2025). **(3)** induce cell cycle arrest by blocking G_0_/G_1_ and G_2_/M phases (Bose et al., 2025; Fauser et al., 2013). **(4)** Alter signalling pathways linked to cancer, including the EGFR-associated pathway (Bose et al., 2025; Sheela et al., 2019), the Rho-associated kinase pathway (Lappano et al., 2017), the PI3K/AKT signalling pathway (Bose et al., 2025; Buva et al., 2024), and and the expression of cancer-associated miRNA (Bose et al., 2025; Verma et al., 2020). **(5)** Reduce pro-inflammatory mediators such IL-6, TNF-α, and iNOS to induce an immunomodulatory effect (Ramya et al., 2022)**. (6)** Prevent metastasis/EMT by inhibiting EMT signalling pathways (Bose et al., 2025) and stemness by reducing the overexpression of the proto-oncogene c MYC (Das et al., 2019; Sandhya Sorra et al., 2019; Sandhya, 2017; Sandhya et al., 2016; Sandhya et al., 2024). Abbreviations: ACSL4, Acyl-CoA Synthetase Long-Chain Family Member 4; AKT, A serine/threonine protein kinase; CAT, Catalase; CD29, Cluster of differentiation 29; CD36, Cluster of differentiation 36; EGFR, Epidermal growth factor receptor; EMT, Epithelial to mesenchymal transition; ERK, Extracellular signal-related kinases; FAS, FS-7-associated surface antigen; GSH, Glutathione; IL6, Interleukin-6; iNOS, Inducible nitric oxide synthase; JUN and c-MYC, Proto-oncogenes; MDA, Malondialdehyde, a production of lipid peroxidation; P21, Cyclin-dependent kinase inhibitor 1; p53, Tumor suppressor gene; PI3K, Phosphatidylinositol 3-kinase; ROS, Reactive Oxygen Species; SOD, Superoxide dismutase; TNFα, Tumor Necrosis Factor alpha.

At the cellular level, LA exerts selective cytotoxicity toward cancer cells by inducing ROS generation (Figure 2), which in turn trigger stress responses and cell death (Bose et al., 2025; Lappano et al., 2017). This ROS-mediated oxidative stress causes mitochondrial dysfunction, mitochondrial membrane potential reduction and pro-apoptotic factor release, and activation of intrinsic apoptosis (Bose et al., 2025). This process involves reduction in the potential of the mitochondrial membrane, release of cytochrome c, followed by caspase-9 and caspase-3 activation, culminating in programmed cell death (Bose et al., 2025). In effect, the MCFAs in CO act in a two-fold manner: they sensitize cancer cells to oxidative challenge, and they may exploit the altered redox balance of tumor cells (Bose et al., 2025). Our previous study demonstrated that CKE has been shown in animal models to decrease oxidative stress by improving superoxide dismutase (SOD), catalase (CAT), and glutathione (GSH)—and decreasing ROS and malondialdehyde (MDA) activity, thereby preventing skin cancer progression (Sandhya Sorra et al., 2019; Sandhya, 2017; Sandhya et al., 2016; Sandhya et al., 2024). Additionally, LA and its monoglyceride derivative, monolaurin, inhibit cancer cells by disrupting their lipid rafts, which are specific membrane domains that are rich in sphingolipids and cholesterol. The disruption of these rafts impairs the receptor-mediated signaling pathways essential for tumor cell proliferation and survival, such as PI3K/AKT, FAS/CD95, VEGF/VEGFR2 and CD44 signalling pathways (Li et al., 2022; Mollinedo and Gajate, 2020).

#### 2.1.2 Apoptosis induction *via* multiple signaling cascades

LA has been demonstrated to cause apoptosis in breast and endometrial cancer cells by phosphorylating EGFR, ERK, c-JUN, and increasing c-FOS expression (Lappano et al., 2017). In colon cancer (HCT-15) cells, LA caused down‐regulation of EGFR expression, contributing to reduced viability through apoptosis (Sheela et al., 2019). Importantly, overexpression of the cyclin-dependent kinase inhibitor p21^Cip1/WAF1^ in a p53-independent manner was observed upon LA exposure, linking to cell-cycle arrest and apoptosis (Lappano et al., 2017). Some studies also report modulation of the BCL-2 family, activation of caspases, and increased BAX/BCL-2 ratio (Figure 2)—though the data for CO derivatives are still limited (Bose et al., 2025).

#### 2.1.3 Proliferation inhibition and cell cycle arrest

Recent *in vitro* studies indicate that both VCO and LA, can suppress tumor‐cell proliferation through induction of cell-cycle arrest. For instance, LA was shown to cause cell-cycle blockade at the G_0_/G_1_ and G_2_/M phases in colon carcinoma cells, alongside elevated reactive-oxygen-species production and glutathione depletion, thereby reducing cell viability (Figure 2) (Bose et al., 2025; Fauser et al., 2013). Moreover, LA treatment in breast and endometrial cancer lines increased expression of the cyclin-dependent-kinase inhibitor p21^Cip1/WAF1^ in a p53-independent manner, which contributes to growth arrest and reduced proliferation (Lappano et al., 2017). A broader review of VCO/LA anticancer mechanisms also notes that VCO and LA inhibit cancer cell viability and proliferation, in part by provoking cell-cycle arrest in preclinical models (Bose et al., 2025). VCO and its fractions FCO and PCO have shown the capability to suppress cancer cell proliferation *in vitro* (e.g., in liver and oral carcinoma lines) with differential efficacy depending on fatty acid composition (Verma et al., 2019).

#### 2.1.4 Modulation of cancer-related signalling pathways

Emerging pre-clinical evidence suggests that VCO and LA influence cancer-related signaling pathways, affecting aspects such as cell cycle transition, apoptosis, growth, oxidative stress response, immune activation, cyclin-dependent kinase activity, EGFR signaling pathways, etc.,. To exert antiproliferative and pro-apoptotic effects. For example, LA has been shown to downregulate the EGFR signalling axis in colon cancer cells, contributing to apoptosis induction (Bose et al., 2025; Sheela et al., 2019). In breast and endometrial cancer models, LA-induced ROS generation caused stress-fiber formation *via* a Rho-associated kinase pathway, phosphorylation of EGFR/ERK/c-JUN, and upregulation of the CDK inhibitor p21^Cip1/WAF1^, all of which ultimately led to apoptosis (Figure 2) (Lappano et al., 2017). LA also alters cancer associated miRNA expression by suppressing metastasis‐related signals (Bose et al., 2025; Verma et al., 2020). Additionally, network pharmacology and molecular docking studies implicate LA in modulating the PI3K/AKT signalling cascade (along with genes such as MAPK1, MMP9 and CXCL8) in oral cancer models (Buva et al., 2024). A broader review of VCO/LA mechanism suggests involvement of cell‐cycle arrest, miRNA modulation, mitochondrial damage, and downstream signalling alterations including EGFR, ERK/JNK/MAPK and possibly PI3K/AKT-mTOR pathways (Figure 2) (Bose et al., 2025).

In 2020, Pruseth et al., reported an *in silico* study on the effects of VCO exposure on cancer-linked gene networks and molecular activity. They analyze that VCO exhibits the capacity to target multiple cancer-associated proteins and pathways, and pathway-enrichment revealed involvement of cell-cycle regulation and apoptosis. Importantly, they have shown that MCFAs from VCO, including myristic acid, LA, caprylic acid, and capric acid, can target almost 17 proteins associated with cancer (Pruseth et al., 2020). Taken together, these findings highlight the potential for VCO/LA to interfere with key oncogenic signalling networks-such as EGFR, ERK/MAPK and PI3K/AKT-to cause apoptosis and inhibit the growth of malignant cells, although further mechanistic and *in-vivo* validation is needed.

#### 2.1.5 Cellular metabolic reprogramming and effect on lipid metabolism

CO-derived fatty acids also modulate cellular metabolism by interfering with *de novo* lipogenesis, a metabolic hallmark of cancer cells. For example, dietary VCO in rats significantly downregulated lipogenic enzyme-encoding transcripts (e.g., FASN, SREBF1) and concomitantly upregulated genes and enzyme activities related to mitochondrial and peroxisomal β-oxidation (e.g., PPARA, carnitine palmitoyltransferase I) relative to other dietary oils—thus reducing *de novo* fatty acid synthesis and enhancing fatty acid catabolism (Arunima and Rajamohan, 2014; Power and Newsholme, 1997).In cancer cell models, LA has been shown to reverse a glycolysis-dominant, stem-like metabolic phenotype towards oxidative phosphorylation (OXPHOS) in chemo-resistant colorectal cancer lines, thereby increasing ROS generation, reducing cell “stemness”, and inducing cell death (Fujiwara-Tani et al., 2025).

Moreover, medium-chain fatty acids such as LA sensitize cancer cells to lipid-peroxidation-driven ferroptosis by up-regulating lipid-metabolism-related transporters (e.g., CD36) and acyl-CoA synthetases (e.g., ACSL4) — linking fatty-acid trafficking and oxidative stress in cancer metabolism (Han et al., 2025). Together these reports indicate that VCO/LA may modulate metabolic rewiring in cells by reducing lipogenesis, increasing fatty-acid oxidation, shifting energy‐production modes, and altering lipid-handling programs—all of which are processes often hijacked in tumor cells.

#### 2.1.6 Selectivity toward cancer vs. normal cells and potential for synergism

Preclinical investigations indicate that LA and VCO exhibit selective cytotoxicity toward cancer cells over normal cells, and may synergize with conventional anticancer drugs while reducing treatment-related toxicity *via* nanoparticle encapsulation. For example, medium-chain fatty acids such as LA were shown to sensitize a variety of cancer cell types to ferroptosis—but not normal cells—via upregulation of CD36 and ACSL4 (Mori et al., 2025; Han et al., 2025). Further, a review outlines that VCO and LA not only have direct anticancer activity but also modulate chemotherapy-induced organ toxicity, for instance protecting against oxidative stress in non-malignant tissues (Bose et al., 2025). Moreover, nano-formulations of VCO (for example, in a nanoemulsion of VCO plus a chemotherapeutic agent) have been reported to enhance drug antiproliferative effects and mitigate drug-induced oxidative damage in healthy tissues (Bose et al., 2025). Taken together, these findings suggest VCO/LA could serve as adjunctive agents that are more selective for malignant cells, capable of synergizing with standard therapies, and potentially reducing side effects when delivered in nanoparticle-based systems.

#### 2.1.7 Induction of immunomodulatory effect

Beyond direct cytotoxicity, CO and its derivatives LA exhibit promising immunomodulatory effects through modulation of both innate and adaptive immune responses, enhancing antitumor immunity. In animal models, experimental research has demonstrated that dietary supplementation with CO extract increases immune parameters like antibody titers, lymphocyte proliferation, and macrophage phagocytic activity, indicating humoral and cellular immunity activation (Usmani et al., 2024). In in vitro model, VCO was reported to increase macrophage phagocytosis of *Staphylococcus aureus*, indicating enhanced innate immune responsiveness (Widianingrum et al., 2019). LA also demonstrates immunoregulatory potential by modulating cytokine expression—reducing pro-inflammatory mediators such as IL-6, TNF-α, and iNOS, while maintaining antioxidant enzyme activity, thereby balancing immune activation and inflammation (Figure 2) (Ramya et al., 2022). Moreover, VCO supplementation was shown to stabilize gut microbiota composition and enhance immune resilience in zebrafish infected with *Aeromonas hydrophila*, supporting its role in host defense modulation (Qosimah et al., 2025). Collectively, these findings suggest that VCO and LA can enhance immune competence and exert anti-inflammatory effects, although further clinical validation is needed to confirm their immunotherapeutic potential in humans.

#### 2.1.8 Inhibition of metastasis/EMT and stemness

Although the literature is still very early, emerging evidence suggests that the VCO and LA may impair key steps of metastasis, such as migration, invasion, and colonization. According to a recent review, in both *in vitro* and *in vivo* models, VCO/LA and their derivatives promote the inhibition of cell motility and metastasis, possibly through suppression of invasion/EMT signaling pathways (Figure 2) (Bose et al., 2025). However, one mechanistic study discovered that, *via* the fatty acid receptor GPR84, LA unexpectedly increased the survival, invasion, and migration of oesophageal squamous cell carcinoma cells (Shen et al., 2025). Another study demonstrated that VCO, FCO, and PCO exhibit modulatory effects on migration and invasion in liver and oral cell lines, although they have not been thoroughly profiled at the molecular level (Verma et al., 2019). Accordingly, VCO/LA may have anti-metastatic potential by down-regulating motility-related signaling pathways, but evidence is inconsistent and varies based on factors like cell type and concentration. Further targeted studies on migration assays, matrix-metalloproteinase activity, and animal models are required to determine if VCO/LA inhibit or potentially promote tumor spread. Additionally, in a murine skin-carcinogenesis model induced by 7,12-Dimethylbenz[a]anthracene/12-O-tetradecanoyl-phorbol-13-acetate (DMBA/TPA), treatment with CKE significantly suppressed overexpression of the oncogene c-MYC—a known driver of cancer-stem-cell (CSC) “stemness” traits (Das et al., 2019; Sandhya Sorra et al., 2019; Sandhya, 2017; Sandhya et al., 2016; Sandhya et al., 2024)— thereby implicating an anti-stemness mechanism that contributes to the prevention of skin tumor progression.

**FIGURE 3:**
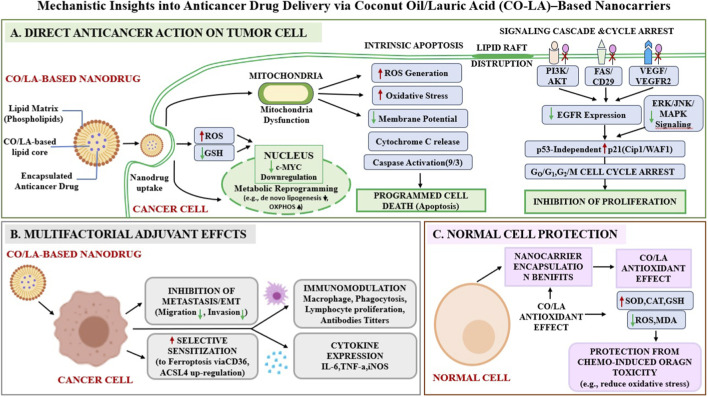
Schematic illustration of the mechanistic pathways underlying the anticancer activity of anticancer drug-loaded Coconut Oil/Lauric Acid (CO-LA)–based nanocarriers in cancer therapy. **(A)** Direct Anticancer Action on Tumor Cell: Highlights the intracellular mechanisms within a tumor cell, including mitochondrial dysfunction (increased ROS, decreased membrane potential, and cytochrome c release) leading to intrinsic apoptosis. It also depicts the disruption of lipid rafts, which inhibits key signaling cascades (PI3K/AKT, FAS/CD29, VEGF, EGFR, ERK, JUN, MAPK) and triggers cell cycle arrest (G_0_/G_1_ and G_2_/M) *via* p53 independent p21^Cip1/WAF1^ upregulation. Metabolic effects, such as reduced *de novo* lipogenesis *via* OXPHOS and c-MYC gene downregulation, are also shown. **(B)** Multifactorial Adjuvant Effects: Demonstrates the systemic benefits of the nanocarrier, including the inhibition of metastasis/EMT and selective sensitization to ferroptosis *via* CD36 and ACSL4. It highlights immunomodulatory effects (increased macrophage/lymphocyte activity and cytokine regulation *via* IL-6, TNF-α, iNOS). **(C)** Normal Cell Protection: Depicts the protective effect on normal cells, where the antioxidant properties of CO (increased SOD, CAT, GSH and decreased ROS, MDA) mitigate chemotherapy-induced oxidative stress and organ toxicity. Abbreviations: ACSL4, Acyl-CoA Synthetase Long-Chain Family Member 4; AKT, A serine/threonine protein kinase; CAT, Catalase; CD29, Cluster of differentiation 29; CD36, Cluster of differentiation 36; EGFR, Epidermal growth factor receptor; EMT, Epithelial to mesenchymal transition; ERK, Extracellular signal-related kinases; FAS, FS-7-associated surface antigen; GSH, Glutathione; IL6, Interleukin-6; iNOS, Inducible nitric oxide synthase; JUN and c-MYC, Proto-oncogenes; MDA, Malondialdehyde, a production of lipid peroxidation; OXPHOS, Oxidative phosphorylation; P21, Cyclin-dependent kinase inhibitor 1; p53, Tumor suppressor gene; PI3K, Phosphatidylinositol 3-kinase; ROS, Reactive Oxygen Species; SOD, Superoxide dismutase; TNFα, Tumor Necrosis Factor alpha; VEGF, Vascular endothelial growth factor.

These combined mechanisms of action highlight the potential of CO/LA-based systems as delivery matrices or adjuvants in anticancer nanodrug formulations. Even so, using CO as a lipid matrix to encapsulate an anticancer drug may improve its therapeutic efficacy by demonstrating a dual effect in the treatment of cancer: the anticancer drug targets cancer cells, while the antioxidant effect of CO protects normal cells from the drug’s toxicity without shielding cancer cells. Importantly, the inherent biocompatibility, bioavailability, and low toxicity of CO make it a promising lipid carrier for anticancer drugs. When employed in nanodrug formulations—such as nanoemulsion, solid lipid nanoparticles, or liposomes—CO/LA not only enhances drug solubility and stability but may also synergistically augment anticancer efficacy through its intrinsic biological activity. Thus, understanding the possible mechanistic pathways of CO’s anticancer action provides a rationale for its integration into advanced nanocarrier systems aimed at improving the therapeutic index of conventional chemotherapeutic agents. Figure 3 shows a schematic representation of the molecular and cellular mechanisms underlying the anticancer activity of anticancer drug-loaded CO/LA-based nanocarriers in cancer therapy.

## 3 Coconut oil-based nanodrug (CO-ND) formulation for drug delivery and its potential in reducing drug-mediated toxicity

CO and LA have been increasingly incorporated into lipid-based nanocarriers, such as SLNs, NLCs, and nanoemulsion to enhance drug solubility and loading, improve physical stability, and optimize drug release. Particularly, the lipid-rich nature of CO provides a matrix for encapsulating or dissolving lipophilic anticancer agents, improving solubility and bioavailability. LA, being amphiphilic, contributes to the stabilization of the lipid matrix and facilitates controlled drug release by influencing the crystallinity and fluidity of the lipid core (M et al., 2024). Moreover, it has been observed that the surface modification of superparamagnetic iron oxide nanoparticles (SPION) by coating them with LA enhances higher rate of anticancer drug release, indicating enhanced biocompatibility of these nanoparticles, thereby improving the intracellular delivery of chemotherapeutic drugs (Yotsomnuk et al., 2025). Furthermore, LA has been studied not only for nutritional/metabolic effects, but increasingly for biomedical applications—either as a bioactive compound itself or as a component of lipid-based nanocarriers (M et al., 2024). In the context of anticancer therapy, the dual challenge is (i) achieving enhanced delivery and efficacy of anticancer drugs (many of which are poorly soluble or display high off-target toxicity), and (ii) reducing the toxicity induced by these drugs in healthy tissues. Thus, CO/LA-based nano formulation offer a promising strategy that addresses both these aspects: vehicle/delivery enhancement + intrinsic bioactivity/protective effects. Below is an example of a few research that demonstrate how CO/VCO/LA-based nanoemulsion/NLCs/micelles containing anticancer medications can be designed to achieve nanometer-scale droplet sizes, stable dispersions, and improved therapeutic efficacy for these medications and drug development.

### 3.1 Metformin–loaded CO nanoemulsion

Metformin (MTF) is an oral antihyperglycemic agent widely used in the management of type 2 diabetes mellitus, was approved by Food and Drug Administration in 1994 (Bailey, 2017; Alotaibi et al., 2023). It primarily acts *via* triggering AMP-activated protein kinase (AMPK), which improves peripheral glucose absorption and reduces hepatic gluconeogenesis (Rena et al., 2017). In addition to its antidiabetic effects, metformin has shown potential anticancer activity through inhibition of the mTOR pathway, induction of cell cycle arrest, and reduction of circulating insulin and IGF-1 levels, which may limit tumor growth. These effects occur through direct mechanisms like AMPK activation and indirect mechanisms, such as reducing the levels of insulin and other growth factors that promote cancer cell proliferation (Alotaibi et al., 2023; Rena et al., 2017; Lei et al., 2017).

**FIGURE 4:**
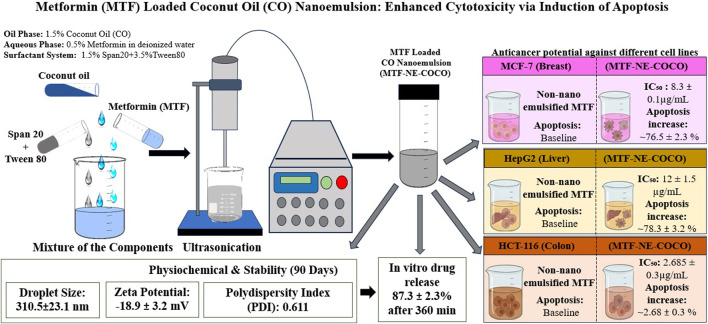
Schematic representation of metformin loaded coconut oil (CO)-based nanoemulsion. The nanoemulsion, prepared by ultrasonication, exhibited a mean droplet size of ∼310 nm, negative zeta potential, sustained drug release, and good stability. MTF-NE COCO showed enhanced cytotoxicity and apoptosis induction in MCF-7, HepG2, and HCT-116 cells compared with free Metformin, indicating improved anticancer efficacy lines (Alotaibi et al., 2023).

In a recent investigation, researchers formulated a nanoemulsion using Metformin loaded into CO (MTF-NE COCO) and assessed its anticancer potential against different cancer cell lines (Alotaibi et al., 2023). Figure 4 shows a schematic of a CO-based nanoemulsion loaded with MTF. The nanoemulsion was prepared *via* an ultrasonication method using 1.5% Span 20, 3.5% Tween 80, 1.5% CO and 0.5% metformin in deionized water, and characterized for droplet size (310.5 ± 23.1 nm), zeta potential (−18.9 ± 3.2 mV), and polydispersity index (PDI) (0.611), *in vitro* drug release (87.3% ± 2.3% after 360 min), and stability droplet size, zeta potential and stability (90 days). The resulting formulation demonstrated significantly improved cytotoxicity against three human cancer cell lines: MCF-7 (breast), HepG2 (liver) and HCT-116 (colon), with IC_50_ values of 8.3 ± 0.1 μg/mL, 12 ± 1.5 μg/mL and 2.685 ± 0.3 μg/mL, respectively—markedly lower compared to non-nanoemulsified metformin. The authors also reported an apoptosis rate increase of ∼76.5 ± 2.3% in MCF-7, ∼78.3 ± 3.2% in HepG2 cells, and ∼2.68 ± 0.33% HCT-116 cells after treatment with the nanoemulsion. Their results suggest that loading metformin into a CO nanoemulsion substantially enhances its antiproliferative efficacy across multiple cancer-cell types.

### 3.2 Methotrexate-loaded CO nanoemulsion

Methotrexate (MTX) is an antimetabolite and antifolate drug used in the treatment of various cancers, including leukemia, lymphoma, and breast cancer, as well as autoimmune diseases such as rheumatoid arthritis (Hanoodi et al., 2025). However, a high dose of MTX may result in numerous serious side effects. Specifically, acute and chronic neurotoxicity can be caused by MTX (Watanabe et al., 2018). According to Howard et al. (2016) and Saygin et al. (2016), MTX can cause lung fibroblasts and alveolitis, making it pulmonary toxic. MTX operates by blocking the enzyme dihydrofolate reductase (DHFR), hence hindering the production of tetrahydrofolate, a critical cofactor for DNA and RNA synthesis (Wong and Choi, 2015). This inhibition results in compromised cellular replication, especially in rapidly proliferating cancer cells. By interfering with these essential metabolic processes, MTX also triggers apoptosis (Howard et al., 2016; Kozminski et al., 2020).

**FIGURE 5:**
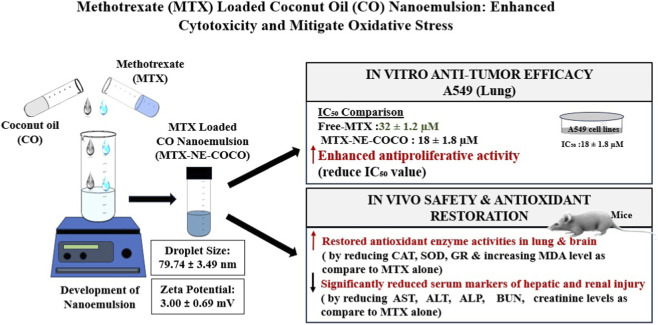
Schematic representation of methotrexate loaded CO-based nanoemulsion. The formulation exhibited a nanoscale droplet size (∼79.7 nm) with stable surface charge and enhanced antiproliferative activity against A549 lung cancer cells compared to free methotrexate (Alkhatib et al., 2020). *In vivo* studies demonstrated reduced oxidative stress, improved antioxidant defense, and decreased hepatorenal toxicity, highlighting the therapeutic advantage of the nanoemulsion system (Alyamani et al., 2020).

One recent study investigated a nanoemulsion formulation of MTX in CO (MTX-NE COCO) in order to enhance anti-tumor efficacy while reducing oxidative stress–related side-effects (Alkhatib et al., 2020). Figure 5 shows a schematic illustration of a CO-based nanoemulsion loaded with MTX. In the experimental protocol, MTX-NE COCO was prepared by dissolving MTX in the coconut-oil-based nanoemulsion and characterized: the droplet size (z-average) was 79.74 ± 3.49 nm (compared with 64.80 ± 3.34 nm for the blank nanoemulsion formulation (NE)) and the zeta potential was modest (3.00 ± 0.69 mV) for the drug-loaded NE. In in vitro studies using A549 non-small cell lung cancer cells, the MTX-NE COCO exhibited a lower IC_50_ (18 ± 1.8 µM) compared with free MTX (32 ± 1.2 µM), indicating enhanced antiproliferative activity. In in vivo experiments in mice, the formulation also attenuated MTX-induced oxidative stress: antioxidant enzymes such as catalase, superoxide dismutase and glutathione reductase in lung and brain tissues were elevated while MDA levels were reduced compared to MTX alone. The authors conclude that incorporating MTX into a CO nanoemulsion improves its cytotoxic efficacy and mitigates oxidative-stress–mediated toxicities in non-target tissues.

Another study, A CO nanoemulsion co-administered with MTX in mouse models significantly reduced serum markers of hepatic and renal injury (AST, ALT, ALP, BUN, creatinine). It also restored antioxidant enzyme activities (CAT, SOD) and reduced lipid peroxidation (MDA), compared to MTX alone (Alyamani et al., 2020).

### 3.3 *Ficus deltoidea* extract-loaded VCO nanostructured lipid carrier

*Ficus deltoidea* is a medicinal plant known for its bioactive compounds, including flavonoids, phenolic acids, triterpenoids, and alkaloids, which contribute to its pharmacological properties (Abrahim et al., 2018; Ashraf et al., 2021; Bunawan et al., 2014). *In vitro* studies reveal that its extract has significant cytotoxic and anti-proliferative effects against various cancer cell lines, including breast, colon, prostate, and cervical cancers, primarily through apoptosis induction and modulation of apoptotic-related proteins such as BAX, BCL-2, and caspases (Abolmaesoomi et al., 2019; Permatasari et al., 2026; Hanafi et al., 2017). The extract also displays strong antioxidant capacity, aiding in cancer prevention while exhibiting selective toxicity against cancer cells and minimal cytotoxicity to normal cells (Abrahim et al., 2018; Ashraf et al., 2021; Permatasari et al., 2026; Al-Koshab et al., 2020), suggesting its potential as a safe anticancer agent for alternative therapies.

A study on *F. deltoidea* extract-loaded VCO NLCs was conducted to enhance the delivery and anticancer potential of the plant’s bioactive compounds (Azmi et al., 2020). The NLCs were prepared using a hot high-shear homogenization technique followed by ultrasonication, where VCO was employed as the liquid lipid and combined with a suitable solid lipid to create a stable nanostructured matrix. The *F. deltoidea* extract was incorporated into the lipid phase prior to emulsification to maximize encapsulation efficiency. The resulting NLCs were evaluated for particle size, PDI, zeta potential, encapsulation effectiveness, and *in vitro* release behaviour. The formulation demonstrated a mean particle size in the nanometre (<200 nm) range with narrow size distribution (PDI <0.3), indicating good homogeneity, and a negative zeta potential suggesting colloidal stability. High encapsulation efficiency was achieved, attributed to the affinity of the phytochemical constituents for the lipid matrix (Azmi et al., 2020). *In vitro* release studies showed a sustained release profile compared to the free extract, while antioxidant activity was preserved after encapsulation, confirming the protective role of the NLCs system (Izza et al., 2022; Huguet-Casquero et al., 2020). These findings indicate that CO-based NLCs are an effective delivery system for enhancing the stability and potential therapeutic performance of *F. deltoidea* extracts.

### 3.4 Doxorubicin-loaded LA-based gold (Au) nanocrystals

Doxorubicin (DOX) is an anthracycline anticancer drug widely used in the treatment of solid tumors (breast, ovarian, lung, bladder, thyroid, sarcomas) and hematological malignancies (leukemia, lymphoma) (Sinha et al., 2025; van der Zanden et al., 2021; Johnson-A et al., 2025). Its anticancer activity is mainly because of DNA intercalation, topoisomerase II inhibition, and reactive oxygen species production, which causes cancer cells to undergo apoptosis (van der Zanden et al., 2021). However, it has dose-dependent toxicities, with cardiotoxicity being the most severe and potentially irreversible, alongside other adverse effects like myelosuppression, gastrointestinal toxicity, and alopecia (van der Zanden et al., 2021; Johnson-A et al., 2025).

In a study, multifunctional gold nanocrystals (AuNCs) co-modified with LA and tumor-targeting aptamers were successfully developed as an advanced theranostic platform for DOX delivery (Sun et al., 2023). Physicochemical characterization confirmed that the dual-functionalized AuNCs preserved uniform nanoscale size and excellent colloidal stability under under physiological circumstances, making them appropriate for systemic administration. LA modification markedly increased the hydrophobic surface area of AuNCs, resulting in significantly higher DOX loading efficiency and improved drug retention compared with unmodified nanoparticles. The presence of tumor-specific aptamers enabled active targeting and selective cellular uptake in receptor-overexpressing cancer cells, leading to enhanced intracellular DOX accumulation and minimized off-target effects. *In vitro* anticancer studies demonstrated superior cytotoxicity of DOX-loaded Apt–LA–AuNCs relative to free DOX, driven by efficient endocytosis and sustained drug release. Furthermore, near-infrared laser irradiation activated the photothermal properties of AuNCs, generating localized hyperthermia that accelerated DOX release and induced additional tumor cell damage, producing a synergistic chemo–photothermal therapeutic effect. Collectively, these results highlight the effectiveness of LA–and aptamer-modified AuNCs as a multifunctional nanoplatform with enhanced drug loading, targeted delivery, and amplified anticancer efficacy through combined chemotherapy, phototherapy, and immune-related mechanisms.

### 3.5 Paclitaxel-loaded LA-O-carboxymethyl chitosan-transferrin micelles

Paclitaxel (PTX) is a potent tetracyclic diterpenoid chemotherapy drug, originally isolated from the bark of *Taxus brevifolia* (Pacific yew), that acts as a microtubule stabilizer to inhibit cancer cell division (Bernabeu et al., 2017; Awosika et al., 2025). Widely used for ovarian, breast, lung, and other solid tumors, it works by inducing mitotic arrest and apoptosis (Awosika et al., 2025). Due to its low yield from natural sources, it is now primarily produced through semi-synthetic methods (Sati et al., 2024).

A previous study demonstrated that PTX was encapsulated into lauric acid-O-carboxymethyl chitosan–transferrin (LA-O-CMCS-Tf) micelles using a solvent-evaporation–induced self-assembly method (Nam et al., 2013). Briefly, PTX was dissolved in a small volume of ethanol and slowly added dropwise to an aqueous solution of LA-O-CMCS-Tf under continuous stirring, allowing hydrophobic interactions between paclitaxel and the LA chains to drive micellization. The organic solvent was subsequently removed under reduced pressure, yielding stable PTX-loaded micelles. Dynamic light scattering analysis showed that the drug-loaded micelles possessed a uniform nanoscale size of approximately 120–150 nm with a narrow polydispersity index, while transmission electron microscopy confirmed a spherical morphology. The encapsulation process markedly increased the aqueous solubility of paclitaxel and achieved high encapsulation efficiency and drug loading. *In vitro* drug-release studies demonstrated a sustained and controlled release behavior compared with free paclitaxel. Furthermore, transferrin conjugation significantly enhanced cellular uptake in transferrin receptor–overexpressing cancer cells, leading to increased cytotoxicity relative to non-targeted micelles. These results demonstrate that LA-O-CMCS-Tf micelles are an effective nanosized carrier for hydrophobic drug delivery, offering improved solubility, controlled release, and site-specific targeted delivery of paclitaxel.

## 4 Pharmacokinetic advantages of coconut oil/lauric acid-based nanodrug (CO/LA-ND) in cancer therapy

The development of lipid-based nanodrug formulations, such as liposomes, SLNs, and NLCs, has become a viable approach in recent years to enhance the therapeutic efficacy and distribution of anticancer drugs, revolutionizing cancer therapy (Mehta et al., 2023; Bayon-Cordero et al., 2019). Among natural lipids, CO, VCO its MCTs, LA, offer multiple, interconnected pharmacokinetic advantages when incorporated into nanocarriers. Lipid-based nanocarriers—particularly SLNs and NLCs—function as protective matrices that modulate drug release profiles, offering several key advantages in chemotherapy (Salvi and Pawar, 2019; Gu et al., 2022; Haider et al., 2020; Wong et al., 2007). These lipid-based nanodrug-delivery systems enhance the bioavailability, drug stability, targeted delivery, particularly for hydrophobic anti-cancer drugs, and therapeutic efficacy of anticancer drugs in addition to addressing significant issues like chemoresistance and specifically targeting aggressive CSCs (Dhanya et al., 2023; Bose et al., 2025; Alotaibi et al., 2023; Alkhatib et al., 2020).

### 4.1 Improved solubility, absorption, and bioavailability

Hydrophobic (lipophilic) anticancer drugs require intravenous administration at high doses to provide therapeutic effects because of their poor oral bioavailability and limited aqueous solubility (often less than 0.1 mg/mL). This leads to systemic toxicity, damage to healthy organs, and potentially limited efficacy due to rapid metabolism or inadequate tumor penetration (Verma et al., 2023; Almawash, 2025). Therefore, the drug’s therapeutic efficacy may enhanced by encapsulating it in CO. CO, which is rich in MCTs like LA, provides a naturally lipophilic medium that enhances hydrophobic medications in the gastrointestinal tract (GIT), leading to the formation of stable, small-globule emulsions (nanoemulsions) that boost bioavailability (Preeti, 2023). Additionally, NLCs in particular employ a combination of liquid and solid lipids to produce an imperfect crystal structure that boosts drug loading capacity and improves stability (Khosa et al., 2018). Furthermore, unlike long-chain triglycerides, MCTs in CO are quickly digested and absorbed straight into the portal vein instead of the lymphatic system (Roopashree et al., 2021). Thus, the enhanced solubility and absorption provided by lipid/CO/LA-based nanoformulations (which encapsulate or dissolve drugs in a lipid matrix) may facilitate greater plasma concentrations of poorly soluble drugs, safeguard drugs from degradation, ensure therapeutic levels are attained with reduced dosages, and consequently diminish systemic toxicity.

In parallel, CO/LA-based nanocarriers can enhance cellular uptake in tumor cells by optimizing dispersion and facilitating interaction with the lipid membrane. These nanocarriers may also interact uniquely with CSCs, although the underlying mechanisms remain poorly understood. CSCs are characterized by altered lipid metabolism, increased membrane fluidity, and elevated expression of lipid transporters, which make them particularly responsive to lipid-based delivery systems (Ali Alghamdi et al., 2024; Lee et al., 2025a; Du and Qin, 2024; Yi et al., 2018; Singh et al., 2024). The amphiphilic nature of LA enhances nanocarrier–cell membrane interactions, facilitating efficient internalization through endocytic pathways (Sahay et al., 2010; Wang et al., 2020). Nanomedicine-based systems exploit these pathways to improve intracellular delivery, reduce the required therapeutic dose, and achieve enhanced treatment outcomes (Verma et al., 2019; Bose et al., 2025; Sahay et al., 2010; Wang et al., 2020). Moreover, emerging evidence indicates that CSCs heavily depend on lipid metabolism to maintain their stemness and survival (Lee et al., 2025a; Du and Qin, 2024; Singh et al., 2024). CO/LA-derived lipids may disrupt these metabolic processes, thereby sensitizing CSCs to anticancer therapies. In addition, lipid-based nanocarriers are highly effective platforms for targeting CSCs due to their biocompatibility, high drug-loading capacity, and flexible surface chemistry. Functionalization with targeting ligands such as hyaluronic acid (for CD44) or aptamers (for CD133) enables active targeting, enhancing cellular uptake while minimizing off-target toxicity (Giordano et al., 2024; Anwar et al., 2024; Kesharwani et al., 2021; Grover et al., 2023; Huang et al., 2020). Following internalization, the sustained release of drugs from CO/LA matrices may further improve therapeutic efficacy by inhibiting efflux pumps and modulating ABC transporter expression, a hallmark of CSC drug resistance (Begicevic and Falasca, 2017; Muriithi et al., 2020; Mandal et al., 2026). Importantly, unlike inert carriers, coconut-derived lipids like VCO and LA exhibit intrinsic anticancer activity in various experimental models, suggesting a potential synergistic effect with loaded drug. These compounds have been shown toinhibit the growth of multiple cancer cell lines (e.g., liver, oral, and breast), through mechanisms such as ROS generation, cell cycle arrest, and apoptosis induction (Verma et al., 2019; Bose et al., 2025; Sheela et al., 2019; Fauser et al., 2013; Lappano et al., 2017). However, it is important to emphasize that direct experimental evidence specifically demonstrating CSC-targeting by CO/LA-based nanocarriers remains limited, with most studies focusing on general cancer cytotoxicity or broader lipid-based delivery systems (Bose et al., 2025; Sheela et al., 2019). This highlights an important area for future research.

### 4.2 Sustained release and prolonged circulation

Nanodrug formulation using CO/LA-based nanocarriers, such as nanoemulsions, SLNs, and NLCs, significantly improve drug delivery by providing controlled, sustained release profiles. These biocompatible, lipid-based systems enhance solubility and stability while maintaining therapeutic plasma concentrations over extended periods (Muller et al., 2000; Mehnert and Mader, 2001; Pardeike et al., 2009). These carriers avoid the high peak plasma concentrations that typically cause severe toxicity, such as cardiotoxicity or gastrointestinal damage, thereby reducing peak systemic drug concentrations. Moreover, the lipidic nature and size (50–1,000 nm) of these nanoparticles allow them to accumulate in tumor tissue *via* the enhanced permeability and retention (EPR) effect, significantly reducing exposure to healthy tissues, thus minimize off-target toxicity (Chaudhary et al., 2024; Waheed et al., 2024). Sustained release not only enhances drug efficacy but also minimizes peak-related toxicities that are common with conventional bolus administration (Siepmann and Siepmann, 2008). Lipid-rich systems also reduce rapid clearance by the reticuloendothelial system (RES), prolonging half-life and enabling more stable pharmacokinetic behaviour (Torchilin, 2005; Alexis et al., 2008). These aspects are critical when designing regimens intended for targeting inherently resistant populations such as CSCs, which may require prolonged exposure to achieve significant cytotoxicity (Dean et al., 2005; Phi et al., 2018).

### 4.3 Decreased systemic toxicity and first-pass metabolism

By lowering first-pass metabolism and limiting systemic toxicity, CO/LA-based nanocarriers provide notable benefits in the delivery of anticancer drugs. When it comes to absorption, LA is different from long-chain lipids. Medium-chain lipid-based nanodrugs restrict significant lymphatic processing and enzymatic degradation because they are more effectively absorbed in the gastrointestinal tract and preferentially delivered into the portal circulation (Bach and Babayan, 1982; Mu et al., 2004; Fricker et al., 2010). During first-pass hepatic metabolism, this absorption pathway decreases unexpected metabolic loss while increasing medication bioavailability.

Crucially, LA and lipids derived from CO have high biocompatibility, are rapidly metabolized, transported directly to the liver, and converted into energy or ketone bodies, rather than being stored in adipose tissue or accumulating in non-target organs (M et al., 2024). These characteristics result in decreased off-target exposure and systemic toxicity when integrated into anticancer nanocarriers, especially hepatotoxicity and immunotoxicity, which are frequently linked to traditional chemotherapy formulations (Puri et al., 2009; Skorzynski et al., 2025; Garcia-Pinel et al., 2019; Namachivayam and Valsala Gopalakrishnan, 2023). CO and LA have anti-inflammatory and antiproliferative properties of its own, which could improve treatment efficacy while safeguarding healthy cells (Bose et al., 2025; Dayrit, 2015; Intahphuak et al., 2010). When combined, these characteristics make nanodrug systems based on CO/LA potential delivery vehicles for strong anticancer drugs with enhanced safety profiles and decreased dose-limiting toxicities. Moreover, lipid-based nanocarriers tend to be biocompatible and biodegradable, minimizing carrier-related immune reactions and off-target toxicity. Such safety profiles support their use as delivery vehicles for high-potency antitumor agents, including those targeting CSCs, without the adverse effects commonly seen with synthetic carriers.

### 4.4 Enhanced targeting and biodistribution

Lipid-based nanodrug delivery systems exhibit superior interactions with biological membranes and enhanced ability to cross physiological barriers, resulting in improved tissue distribution and therapeutic efficiency (Porter et al., 2007; Mohite et al., 2023; Yoon et al., 2013). In this Regards, CO/LA-based nanocarriers enhance cellular uptake in tumor cells by improving dispersion and promoting interaction with the lipid membrane. These nanocarriers primarily utilize endocytic pathways for cellular entry, enabling efficient intracellular delivery while reducing the required therapeutic dose and improving treatment outcomes (Verma et al., 2019; Bose et al., 2025). Importantly, unlike conventional inert carriers, coconut-derived lipids like VCO and LA possess intrinsic anticancer activity. Experimental evidence demonstrates that these lipids exert anticancer effects across multiple cancer types, including liver, oral, and breast cancers as shown in Table 2, through a number of biological processes, including the production of ROS, cell cycle arrest, and activation of apoptosis (Verma et al., 2019; Bose et al., 2025; Sheela et al., 2019; Fauser et al., 2013; Lappano et al., 2017). This intrinsic activity suggests a synergistic interaction between the carrier and the encapsulated drug, enhancing overall therapeutic efficacy.

Targeting CSCs remains a major challenge due to their role in drug resistance and tumor relapse (Phi et al., 2018; Lee et al., 2025b). Nanocarriers can be engineered to selectively target CSCs by functionalizing their surfaces with ligands against CSC-specific markers such as CD44 and CD133, or by exploiting tumor microenvironment features such as the EPR effect for preferential accumulation (Kesharwani et al., 2021; Grover et al., 2023; Huang et al., 2020; Singh et al., 2003; Maeda et al., 2000). These strategies improve drug localization within CSC niches, thereby enhancing retention and intracellular delivery. As a result, nanomedicine approaches are increasingly recognized for their ability to overcome conventional resistance mechanisms associated with CSCs, including drug efflux and cellular quiescence (Liu et al., 2023; Konrad et al., 2017; Daoud et al., 2025). Moreover, the lipophilic nature of CO/LA-based carriers facilitates surface modification and ligand conjugation, further improving CSC homing and uptake, although direct experimental validation specific to CO/LA systems remains limited (He et al., 2016; Priya et al., 2023).

**FIGURE 6:**
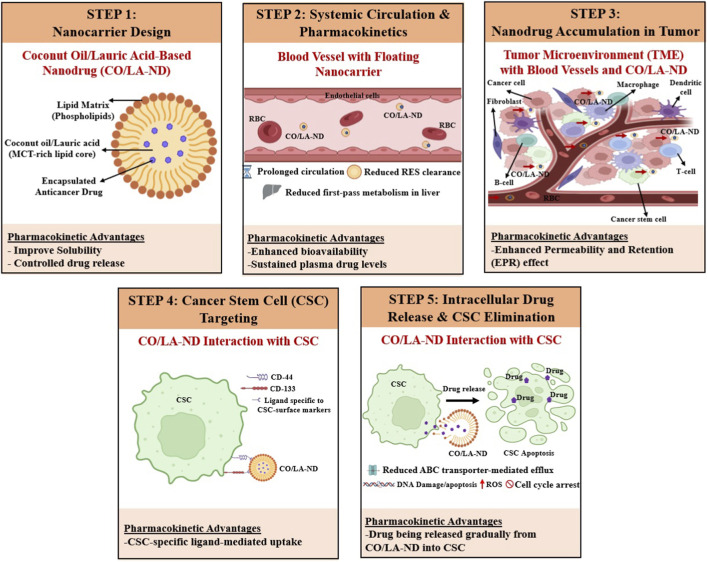
Schematic illustration of coconut oil/Lauric Acid–based nanodrug (CO/LA-ND) design, systemic behavior, tumor accumulation, and cancer stem cell (CSC) targeting with pharmacokinetic advantages as Pharmacological Strategy. **Step 1:** CO/LA-ND formulation comprising a phospholipid shell and a medium-chain triglyceride (MCT)-rich CO/LA lipid core encapsulating an anticancer drug, enabling improved solubility and controlled drug release. **Step 2:** Following systemic administration, CO/LA-ND circulates in the bloodstream with prolonged circulation time, reduced reticuloendothelial system (RES) clearance, and diminished first-pass hepatic metabolism, leading to enhanced bioavailability and sustained plasma drug levels. **Step 3:** CO/LA-ND preferentially accumulates within the tumor microenvironment (TME) *via* the enhanced permeability and retention (EPR) effect, interacting with cancer cells, cancer stem cells (CSCs), immune cells (T-cell and B-cell), macrophages, dendritic cells and fibroblasts surrounding tumor vasculature. **Step 4:** CO/LA-ND selectively targets CSCs through ligand-mediated interactions with CSC surface markers (e.g., CD44, CD133), promoting CSC-specific uptake. **Step 5:** Intracellular drug release from CO/LA-ND within CSCs might results in reduced ABC transporter–mediated drug efflux, increased ROS generation, DNA damage, cell cycle arrest, and apoptosis, ultimately leading to CSC elimination.

Figure 6 schematically illustrates the design and pharmacokinetic behavior CO/LA-based lipid nanocarriers for CSC-Targeted Drug Delivery. In this system, the nanocarrier core is composed of MCT-rich lipid such as CO or LA-derived from CO, forming a lipophilic matrix that enhances drug solubility, stability, and controlled release. Following systemic administration, these nanocarriers exhibit prolonged circulation time and reduced reticuloendothelial system (RES) uptake, facilitating preferential accumulation in tumor tissue *via* EPR effect. Surface functionalization with CSC-specific ligands (e.g., CD44 or CD133 targeting moieties) (Kesharwani et al., 2021; Grover et al., 2023; Huang et al., 2020) promotes selective binding and internalization into CSCs within the TME. Once internalized, sustained drug release ensures prolonged intracellular exposure, enabling the circumvention of ABC transporter-mediated drug efflux and enhancing cytotoxicity against quiescent, drug-resistant CSC populations. Additionally, LA contributes membrane-modulating and pro-apoptotic effects, further sensitizing CSCs to chemotherapeutic agents while minimizing toxicity to normal cells. Collectively, this targeted strategy improves pharmacokinetic selectivity, reduces off-target exposure, and holds promise for suppressing tumor relapse and metastasis. Additionally, LA contributes intrinsic pro-apoptotic and membrane-modulating effects, further sensitizing CSCs to chemotherapeutic drugs while minimizing toxicity to normal cells. Collectively this strategy improves pharmacokinetic selectivity, reduces off-target exposure, and hold promise for suppressing tumor relapse and metastasis (Batlle and Clevers, 2017).

Importantly, CO/LA-ND systems offer several advantages over conventional lipid nanocarriers such as liposomes, SLNs, and NLCs, which primarily function as passive drug delivery vehicles. First, CO/LA-ND systems exhibit a dual functional role, acting both as carriers and as biologically active agents due to the inherent anticancer, anti-inflammatory, and membrane-modulating properties of coconut-derived lipids (Verma et al., 2019; Bose et al., 2025; Sheela et al., 2019; Fauser et al., 2013; Lappano et al., 2017; Dayrit, 2015; Intahphuak et al., 2010). Second, they display a distinct pharmacokinetic profile characterized by rapid digestion, portal vein absorption, and reduced dependence on lymphatic transport, which may lead to faster and more predictable systemic availability compared to long-chain lipid-based systems (Roopashree et al., 2021; Bach and Babayan, 1982; Mu et al., 2004; Fricker et al., 2010). Third, these systems enable simplified and cost-effective formulation approach by leveraging natural, biocompatible lipids that require fewer synthetic surfactants and simpler manufacturing processes like high-shear homogenization or ultrasound. These systems form stable nanocarriers with high encapsulation efficiency for hydrophobic drugs (Dhanya et al., 2023; Salvi and Pawar, 2019; Gu et al., 2022; Gani and Benjakul, 2018). Finally, their ability to modulate tumor metabolism, enhance CSC sensitivity, improve drug solubilization, and provide sustained release positions them as multifunctional therapeutic platforms rather than purely passive carriers (Lee et al., 2025a; Du and Qin, 2024; Yi et al., 2018; Singh et al., 2024; Li H. et al., 2020). Overall, the innovation of CO/LA-ND delivery systems lies not in replacing existing lipid nanocarriers, but in offering a mechanistically distinct, bioactive, and translationally promising alternative for targeted cancer therapy.

### 4.5 Safety and biocompatibility

CO is generally recognized as safe (GRAS) by the U.S. FDA for use in foods, including direct addition to food products for human consumption, based on its long history of common use and safety data (Burnett et al., 2011). Specifically in its virgin form (VCO), CO has emerged as a functional food oil with potential therapeutic benefits due to its rich content of bioactive compounds, particularly MCFAs. VCO contains a high proportion of MCFAs such as LA, which has been associated with several pharmacological activities and health-related effects (Bose et al., 2025; Prasanna et al., 2024). Because of its fatty acid profile and biocompatibility, CO and its derivatives are being investigated as biocompatible, nontoxic carriers for the delivery of therapeutic agents in functional and drug delivery systems (Rodrigues et al., 2024). LA itself exhibits biological effects that may synergize with encapsulated anticancer drugs. Studies indicate that LA can modulate cancer cell survival pathways—such as downregulating EGFR signaling, induce ROS-mediated apoptosis in cancer cells, *etc.*, as shown in Figure 2 (Bose et al., 2025; Sheela et al., 2019; Mori et al., 2025; Fauser et al., 2013; Lappano et al., 2017). Although these effects are not exclusive to CSCs, such intrinsic bioactivity may enhance cytotoxic pressure on tumor cells, including stem-like subpopulations that are typically resistant to apoptosis and standard therapies (Phi et al., 2018). LA may alter pharmacokinetics (such as increased cellular absorption and membrane partitioning) and pharmacodynamics, increasing the susceptibility of resistant tumor subpopulations to therapeutic medicines (Torchilin, 2014; Rajpoot, 2019).

Emerging evidence indicates that incorporation of VCO/LA into formulations containing standard chemotherapeutics [e.g., Metformin (Alotaibi et al., 2023), methotrexate (Alkhatib et al., 2020), doxorubicin (Sun et al., 2023), and Paclitaxel (Nam et al., 2013)] enhances antiproliferative efficacy to the free drug, while simultaneously attenuating oxidative stress and other toxicity markers. This dual action may be attributed to the biochemical composition of coconut derivatives, including medium-chain fatty acids, triglycerides, and phenolic compounds, which confer antioxidative and anti-inflammatory properties. These components may protect healthy tissues from chemotherapy-induced ROS and inflammatory damage while preserving or enhancing cytotoxic effects in cancer cells.

Collectively, these findings suggest that CO/LA-ND systems can simultaneously enhance anticancer efficacy, improve therapeutic selectivity, and mitigate chemotherapy-induced side effects. By integrating drug delivery, bioactive lipid synergy, and nanoscale targeting advantages, such formulations represent a promising strategy for improving the safety and effectiveness of anticancer therapies.

In comparison with conventional nanodrug delivery systems that primarily function as passive carriers, CO/LA-NDs provide several distinctive advantages that justify their consideration as a central focus of this review. The MCTs present in CO—particularly LA—serve not only as a lipid matrix for drug encapsulation but also as biologically active components that can enhance therapeutic outcomes. These lipids improve the solubilization and absorption of poorly water-soluble anticancer drugs, enable sustained drug release, and promote favorable pharmacokinetic behavior such as prolonged circulation and improved tumor accumulation through the EPR effect. Importantly, unlike many conventional inert nanocarriers, coconut-derived lipids possess intrinsic biological activities, including pro-apoptotic, anti-proliferative, and membrane-modulating effects, which may synergize with encapsulated chemotherapeutic agents. This dual role—acting simultaneously as a delivery matrix and a bioactive therapeutic adjuvant—distinguishes CO/LA-ND systems from traditional lipid or polymeric carriers. Consequently, CO/LA-NDs represent a multifunctional nanotherapeutic platform capable of improving drug bioavailability, enhancing targeting of resistant tumor subpopulations such as cancer stem cells, and potentially reducing systemic toxicity.

## 5 Conclusion

This review highlights the emerging potential of CO and its derivatives VCO and LA–based lipid nanocarriers to encapsulate anticancer drugs to construct CO/LA-ND formulations as versatile and biocompatible platforms for anticancer drug delivery. Collectively, Incorporating CO, VCO, and LA into nanodrug formulations confers several pharmacokinetic advantages—enhanced solubility and bioavailability, sustained release, improved biodistribution, prolonged circulation, reduced first-pass metabolism, and enhanced tumor accumulation *via* the EPR effect—underscore their capacity to optimize anticancer drug performance while lowering systemic exposure to healthy tissues (Dhanya et al., 2023; Jadhav and Annapure, 2023). At the same time, these lipid-based nanocarriers may significantly mitigate drug-mediated toxicities, including oxidative stress, hepatotoxicity, nephrotoxicity, pulmonary toxicity, and cardiotoxicity, which are major limitations of current chemotherapy regimens (Yazbeck et al., 2022; Postow et al., 2018; Sandhya Sorra et al., 2019; Sandhya, 2017; Sandhya et al., 2016; Sandhya et al., 2024; Famurewa et al., 2017; Alyamani et al., 2020; Skorzynski et al., 2025; Namachivayam and Valsala Gopalakrishnan, 2023). Importantly, unlike inert carriers, coconut-derived lipids possess intrinsic bioactivity, including pro-apoptotic, antioxidant, anti-inflammatory, and membrane-modulating effects, which may synergize with encapsulated drugs to enhance cytotoxicity in cancer cells while protecting normal tissues (Bose et al., 2025; Ramya et al., 2022; Sheela et al., 2019; Mori et al., 2025; Fauser et al., 2013; Lappano et al., 2017; Han et al., 2025). In this regard, we also found that CKE possesses significant antioxidant capacity and prevented tumor formation in carcinogen-induced skin cancer models by mitigating oxidative stress and c-MYC proto-oncogene overexpression (Sandhya Sorra et al., 2019; Sandhya, 2017; Sandhya et al., 2016; Sandhya et al., 2024). Additionally, we also found that lauric acid, a fatty acid originating bioactive compound, plays a significant role in CKE (Sandhya et al., 2024). Therefore, here we suggest that using CO or VCO/LA to encapsulate Pt-AD in nanoform, may enhance a dual effect in the treatment of cancer: the anticancer drug targets cancer cells, while antioxidant effect of CKE shields normal cells from the toxicity effects of Pt without protecting cancer cells. Furthermore, c-MYC proto-oncogene is a key player in cancer progression, increases pluripotency and tumorigenicity (Sandhya et al., 2024; Knoepfler, 2009; Greulich et al., 2000). This gene causes cells to grow aggressively by interfering with cell division, proliferation, and apoptosis (Sandhya et al., 2024; Dhanasekaran et al., 2022; Kaczmarek et al., 1985; Zindy et al., 1998; Amati and Land, 1994; Schlagbauer-Wadl et al., 1999; Marconi et al., 2022; Kreuzaler et al., 2019; Zimmerli et al., 2022). Researchers are therefore looking into this gene as a potential target for cancer treatment (Duffy et al., 2021; Wang et al., 2021; Llombart and Mansour, 2022; Chen et al., 2018). Previous, we found CSCs maintain self-renewal capacity by c-MYC-mediated HIF-2α stemness pathway *via* NANOG and SOX-2 (Das et al., 2019). Therefore, by synthesizing CKE-Pt-AD conjugated nanoparticles, we may able to also target the c-MYC mediated stemness pathway in cancer cells. Furthermore, emerging evidence further suggests that CO/LA-based nanocarriers may play a critical role in addressing chemoresistance and tumor recurrence by improving drug retention and exposure in resistant tumor subpopulations, including CSCs. Although direct CSC-targeting studies using CO/LA-based systems remain limited, the physicochemical properties of these lipid matrices—combined with surface functionalization strategies—provide a strong rationale for their application in CSC-directed therapies. Overall, CO/LA-ND systems represent a promising, multifunctional, and translationally relevant approach for improving the safety, selectivity, and efficacy of anticancer treatments. In addition, these carriers offer a platform adaptable to CSC-targeted strategies, addressing one of the core barriers to durable cancer therapy by potentially improving drug delivery to resistant, stem-like tumor cells. Continued research integrating lipid nanocarriers with CSC-specific targeting mechanisms may further unlock their promise for next-generation anticancer therapeutics.

## 6 Future directions

Despite the encouraging preclinical evidence, several key areas warrant further investigation to advance CO/LA-ND systems toward clinical translation. First, comprehensive pharmacokinetic and biodistribution studies in relevant animal models are needed to quantitatively compare CO/LA-ND formulations with existing lipid-based and polymeric nanocarriers. Such studies should evaluate long-term accumulation, metabolic fate, and potential off-target effects following repeated administration. Second, while *in vitro* and *in vivo* data support reduced toxicity and enhanced efficacy, well-designed preclinical safety studies—including chronic toxicity, immunogenicity, and reproductive toxicity—are essential to establish robust safety profiles. This is particularly important for multifunctional and surface-modified nanocarriers intended for systemic and long-term use. Third, future research should focus on the rational design of CO/LA-based nanocarriers with active targeting capabilities, especially toward CSCs. Incorporation of ligands specific to CSC markers (e.g., CD44, CD133, ALDH1) and tumor microenvironment–responsive elements could further enhance selectivity, overcome drug efflux mechanisms, and suppress tumor relapse and metastasis. Direct experimental validation of CSC eradication using CO/LA-ND systems remains a critical gap in the current literature. Fourth, mechanistic studies are needed to better elucidate the synergistic interactions between coconut-derived lipids and anticancer drugs at the molecular level, including their effects on membrane dynamics, redox signaling, apoptotic pathways, and drug transporter activity. Understanding these interactions will facilitate optimized formulation design and dose reduction strategies. Finally, translational and clinical research efforts—including scalable manufacturing, formulation standardization, and early-phase clinical trials—are essential to determine the real-world feasibility of CO/LA-ND systems. Given their GRAS status, biodegradability, and intrinsic bioactivity, coconut-derived lipid nanocarriers hold strong promise as next-generation platforms for safer, more effective, and patient-friendly cancer therapies.
